# Artificial intelligence in colorectal surgery multidisciplinary team approach—From innovation to application

**DOI:** 10.14814/phy2.70319

**Published:** 2025-04-26

**Authors:** Ali Murtada, Fatima Kayali, Matti Jubouri, Samuel N. S. Ghattas, Samuel S. S. Rezk, Feroze Ahmed Mir, Ian Williams, Mohamad Bashir, Damian M. Bailey

**Affiliations:** ^1^ Department of General Surgery Ysbyty Glan Clwyd Rhyl UK; ^2^ University Hospitals Sussex Worthing West Sussex UK; ^3^ Hull York Medical School, University of York York UK; ^4^ Department of Vascular Surgery University Hospital of Wales Cardiff UK; ^5^ Neurovascular Research Laboratory, Faculty of Life Sciences and Education University of South Wales Pontypridd UK

**Keywords:** artificial intelligence, clinical decision‐making, colorectal Cancer, colorectal surgery, multi‐disciplinary team

## Abstract

Artificial intelligence (AI) has played a novel role in aiding healthcare system functions and enhancing the patient experience. Multidisciplinary teams (MDT) have become an integral part of disease and management planning, especially with the rising number of our aging population and the paucity of sufficient resources. The incorporation of MDTs facilitates a holistic approach to patient care, encompassing the physical, psychological, and social needs of patients and their families. Particularly with the growing number of colorectal cancer diagnoses, notably among the younger populations, the utilization of AI in the colorectal MDT holds great potential value. The ability to enhance the quality of these interdisciplinary discussions will likely reflect on improving holistic patient‐centered care and reducing the numbers of late or misdiagnosis. In addition, the incorporation of AI into these meetings will aid in reducing the workload on healthcare professionals and reduce the financial burden on pressurized healthcare systems. This narrative review article explores the role of AI in the colorectal surgery MDT, its drawbacks, and its merits.

## 1 INTRODUCTION

Artificial Intelligence (AI) has demonstrated its capacity to predict surgical outcomes and assess personalized patient risks through data analysis. Additionally, AI's image recognition technology has adeptly identified fractures and detected breast cancer. In supervised surgical settings, robots have proficiently executed suturing and bowel anastomosis in controlled environments. AI holds the potential for integration into surgical services, encompassing diagnosis, treatment, and provision of decision support for surgeons regarding patient risks.

AI comprises four principal subfields: (Hashimoto et al., 2018) machine learning, (Pillay et al., 2016) artificial neural networks, (Prades et al., 2015) natural language processing, and (Pan et al., 2015) computer vision (Hashimoto et al., 2018). The contemporary and future applications of AI within surgical practice, including big data analytics and clinical decision support systems, were expounded upon. The anticipated implications of AI for surgeons and their role in advancing the technology to maximize clinical effectiveness and outcomes are key facets of AI's potential in surgical practice. Suggestions have also been articulated for intraoperative AI‐enhanced surgery and the integration of AI into robotic platforms.

However, the integration of AI within the multidisciplinary team (MDT) approach and its potential influence on clinical decision‐making from the preoperative phase to the development of tailored treatment algorithms, as well as its support for clinical decision‐making, assessment of complication risk, prediction of surgical outcomes and survival, remains relatively obscure (Pillay et al., 2016). Data on these aspects are limited, and AI's application in colorectal surgery is still emerging. Nonetheless, the rapid evolution of technology makes its increasing integration into everyday practice probable. This narrative review article endeavors to navigate and explore this terrain and provide insight into technology‐driven advancements, where scientific and technology‐driven approaches lead clinical practice.

## 2 THE PROS AND CONS OF COLORECTAL MDT

A multidisciplinary approach in colorectal surgery offers several notable advantages. It facilitates a holistic approach to patient care, encompassing the physical, psychological, and social needs of patients and their families. Furthermore, it enables the amalgamation of knowledge and expertise from diverse disciplines, leading to a more comprehensive understanding of intricate healthcare concerns. The involvement of multidisciplinary teams fosters coordinated and uninterrupted care, resulting in heightened patient and family contentment, enhanced quality of life, and even marginal improvements in survival rates for select patient cohorts (Pan et al., 2015; Prades et al., 2015).

Nevertheless, the adoption of multidisciplinary approaches also presents challenges. Effective coordination of care across various locations, personnel, and temporal boundaries is essential to surmount barriers and ensure the efficacy of multidisciplinary approaches. Additionally, it is imperative to discern that not every healthcare undertaking necessitates multidisciplinary teamwork, and its applicability may not be universal across all healthcare scenarios.

**FIGURE 1:**
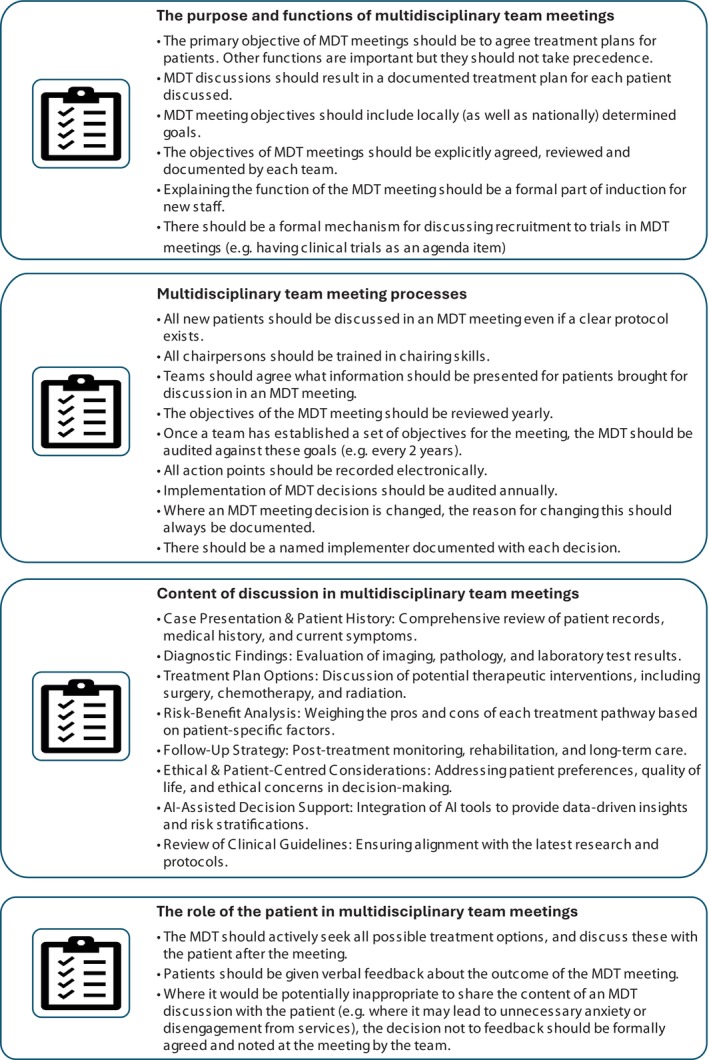
Twenty‐one indications of good practice for an effective multi‐disciplinary meeting by Raine et al. (2014).

The colorectal MDT demonstrates similarities to the interdisciplinary MDT in the context of surgical care and provision. However, divergent conclusions from various studies examining the relationship between MDTs, quality of care, and survival can be attributed to differences in study design, MDT structure, case selection, and diagnoses (Munro et al., 2015; Prades et al., 2015). It is important to emphasize that increasing multidisciplinary does not necessarily guarantee more effective decision‐making and treatment implementation. The efficacy of an MDT is influenced by several factors, including the participation of qualified and effective experts, case selection, access to relevant information, discussion format and structure, leadership, interactions among health professionals, technical resources, and administrative procedures (Raine et al., 2014). The twenty‐one principles of good practice and effective MDTs, illustrated in Figure 1, were created by Raine et al. (2014) in their prospective observational study across London and the North Thames.

There is an increasing trend toward using video‐based multidisciplinary team meetings with regional or national participation. Although the value of this approach is widely recognized, the structure is being reassessed due to resource demands driven by the growing incidence of colorectal cancer, in addition to increased demand on surgical activity and volume, greater involvement of experts, the need for more frequent meetings to ensure timely treatment, evaluation based on advanced diagnostic methods, and the use of increasingly complex treatment algorithms (Vuik et al., 2019).

We now offer more advanced and personalized treatments to patients with increasingly complex cases. However, a 2017 study by Cancer Research UK found that there was not enough time in MDT meetings to discuss more complex patients, with around half of patients being discussed for 2 min or less (Cancer Research UK, 2017). In response, the Independent Cancer Taskforce Report recommended that NHS England encourage providers to allocate specialist time in MDT meetings to cases that do not fit well‐established clinical pathways (Independent Cancer Taskforce Report, 2015).

Within the realm of stringent criteria and systematic evaluation, it is not only advisable but also essential to assess the quality of colorectal MDT forums. Recognizing the absence of standardized quality benchmarks for these gatherings, healthcare systems have prioritized the development of quality assurance and evaluation strategies. The introduction of validated observational instruments to monitor the quality of MDT forums is essential. In the domain of colorectal cancer, Shah and colleagues have published their findings in this area (Shah et al., 2014). They have introduced the “Colorectal Multidisciplinary Team Metric for Observation of Decision‐Making (cMDT‐MODe)”, which is a validated tool designed for the assessment of the quality of colorectal MDTs. This tool represents a modification of implemented quality assessment instruments. Seretis et al. (Seretis et al., 2014) applied the cMDT‐MODe tool in their prospective analysis of 64 cases of colorectal cancer. Seen in Table 1, this tool aided the surgeons in assessing the quality of the MDTs; adequate history was presented (4.4 out of 5), but insufficient radiological and histopathological results were available at the time of discussion (3.9 and 3.8 out of 5, respectively).

**TABLE 1:** Example of Proforma using the Colorectal Multidisciplinary Team Metric for Observation of Decision‐Making (cMDT‐MODe) by Seretis et al. (2014).

Component	Description
Case History	…/5: Fluent case history with salient points clear …/3: Partial case history …/1: No patient history
Radiological information	…/5: Radiological images, with clear information regarding staging and margins …/3: Radiological information from report/account OR partial information about staging/margins …/1: No provision of radiological information
Pathological information	…/5: Histopathological information with concise information regarding resection margins …/3: Histopathological information with some information regarding resection margins …/1: No provision of histopathological information
Contribution of MDT chair	…/5: Leadership enhancing information/presentation/discussion/decision making …/3: Leadership not enhancing information/presentation/discussion/decision making …/1: Leadership impeding information/presentation/discussion/decision making
Psychosocial factors	…/5: Comprehensive knowledge of patient's personal circumstances, social, psychological issues …/3: Vague knowledge of patient's personal circumstances, social, psychological issues …/1: No knowledge of patient's personal circumstances, social, psychological issues
Patient comorbidity	…/5: Comprehensive knowledge of patient's history, performance status and relevant anatomical info …/3: Vague knowledge of patient's history, performance status and relevant anatomical info …/1: No knowledge of patient's history, performance status and relevant anatomical info
Patient views	…/5: Comprehensive knowledge of patient's opinions/wishes regarding treatment …/3: Vague knowledge of patient's opinions/wishes regarding treatment …/1: No knowledge of patient's opinions/wishes regarding treatment
Contribution of members	…/5: Articulate & precise specialty related contribution …/3: Contribution inarticulate or vague …/1: Nil/impedes contribution of others
Final MDT recommendation	Y – clear recommendation about treatment N – no or unclear recommendation

The absence of standardized processes in colorectal MDT and quality assessment tools necessitates the utilization of AI to drive innovation and facilitate essential quality enhancement and implementation efforts.

## 3 AI FOR QUALITY ASSURANCE & COST DRIVERS OF COLORECTAL MDT

Each clinical decision involves an economic component. The cost of providing limited healthcare resources includes the inability to fund alternative therapies, resulting in losing their benefits. While robotics in colorectal surgery offers new potential treatments, assessing their performance compared to the current gold standard is essential and determining if the added cost is justified. A personalized, patient‐centred approach to medicine has led to the utilization of quality‐adjusted life years as the primary means of measuring overall health outcomes, particularly by resource allocators in the United Kingdom. Colorectal surgery interventions are resource‐intensive, and recent trends show a growing economic burden due to increasing annual costs (Stukalin et al., 2022). Despite their weaknesses, health economic models must consider the effect on society, distributional consequences, and the value of gathering more information to reduce economic uncertainty in future analyses.

The integration and amalgamation of cost drivers in colorectal surgery MDT is a dynamic process. In the UK, considerable investment has been directed toward ensuring the efficacy of cancer MDT meetings. It is estimated to cost the National Health Service (NHS) approximately £50 million annually for preparation and a similar amount for attendance time (Taylor et al., 2010). However, the justification for this substantial investment remains unexplored and has not been previously addressed. Data integration into this concept is integral for further innovation and alignment of quality assurance meeting cost‐effectiveness.

The incorporation of AI into the current colorectal MDT will bolster the foundations of quality assurance and streamline cost‐efficiency. Implementing artificial intelligence technologies, including machine learning, natural language processing, and computer vision, will elevate and automate several facets of the quality assurance process. This will empower teams to efficiently address intricate tasks, identify discrepancies, and swiftly make precise, data‐driven decisions. The initiative aims to redress the identified shortcomings in colorectal MDT, providing a platform for change and mandating a pathway to meet unmet clinical needs.

In building a reproducible model for integrating AI into colorectal MDT assessment and quality assurance, it is crucial for AI to deliver value, automate testing, facilitate defect detection, and carry out performance evaluations. To develop an effective AI tool and framework, it is imperative to adhere to AI fundamentals by gathering and preparing data, as well as cleaning and pre‐processing data to remove noise and inconsistencies. The AI model should continuously monitor performance and assess its efficacy in terms of accuracy, speed, and resource utilization. Additionally, the AI needs to adapt parameters or retrain models as necessary. The model should promote collaboration and be governed to bolster clinical decision‐making, presenting predictions and delineating outcomes.

To develop cost estimates, it is necessary to utilize statistical analysis or algorithms to analyze past data and generate representative estimates. In a colorectal MDT, the primary cost driver is the time and services provided by participants (Pillay et al., 2016). Consequently, AI can calculate the average cost per unit of time and service and then apply that average cost to all other services. Nonetheless, there are several limitations to this method of cost modeling. For example, the dataset may not consider external factors such as inflation.

Additionally, an algorithm may encounter difficulty in managing the complex interconnected variables involved in colorectal MDT meetings. Advanced machine learning algorithms can consider intricate, dynamic factors such as inflation and provide more accurate and predictive estimates for future costs (Medeiros et al., 2019). Machine learning can process more data than humans can at any given time. These cost models enable automation—they can be updated in real time. They can be developed without the need for technical experts or data scientists, allowing swift and easy prediction of cost information as part of automated decision‐making. Consequently, this innovative approach to cost modeling has become an indispensable tool for many healthcare providers aiming to enhance current health practices and promote innovation. Table 2 provides an overall summary of the findings in this section.

**TABLE 2:** AI for Quality Assurance & Cost Drivers (Section 3 Summary).

Aspect	Description
Role of AI in Quality Assurance	AI applications for detecting errors, standardizing reporting, and improving diagnostic accuracy.
Automation Benefits	Reduction in manual workload, improved efficiency, and real‐time decision support.
Cost Analysis	Identification of cost drivers, impact on overall MDT (Multidisciplinary Team) expenses.
Challenges	Ethical concerns, data privacy issues, and need for robust validation.
Implementation Considerations	Required infrastructure, training, and integration with existing systems.

## 4 AI FOR MULTI‐CRITERIA DECISION‐MAKING OF COLORECTAL MDT

Decision‐making is a complex mental process that aims to achieve a desired outcome while considering various factors. This process can be influenced by physiological, biological, cultural, and social factors, authority, and risk levels, and can be rational or irrational. Complex decision‐making problems can be addressed using mathematical equations, statistics, economic theories, and computer devices.

Multi‐Criteria Decision Making (MCDM) or Multi‐Criteria Decision Analysis (MCDA) is an accurate decision‐making method (Diaby et al., 2013). MCDM has the potential to minimize conflicts inherent in clinical decision‐making, enhance knowledge, and achieve outcomes that reflect the values of the decision‐makers. While there is no uniformly superior approach or method for applying MCDA, the decision‐making context can guide the selection of the most appropriate method. When combined with AI techniques, MCDM provides powerful tools for making complex decisions in multifaceted situations. These methods aim to rank or prioritize alternatives by aggregating criteria and providing decision‐makers with valuable insights.

These features illustrate multifaceted attributes, such as textural characteristics of a lesion extracted from different image modalities and treatment prescriptions for individual patients. However, limitations on radiomics have been reported. To this effect, MCDM is both an approach and a set of techniques developed in decision theory to aid in problem solving. It is used to address diverse issues, and the selection of the optimal MCDM method varies depending on the source and nature of information used to inform decision making. It also depends on the model the decision makers believe matches their ability.

In multicriteria analysis, a performance matrix or consequence table is commonly used (Diaby et al., 2013). This matrix includes rows describing options and columns describing the options' performance against each criterion. Various methods, such as the weighted sum product and the weighted sum method, are used to aggregate judgments. An overview of this section can be found in Table 3.

**TABLE 3:** AI for Multi‐Criteria Decision‐Making (Section 4 Summary).

Factor	Description
Decision Support Role	AI's ability to analyze multiple variables to optimize MDT decisions.
Predictive Analytics	AI‐driven models for prognosis, treatment planning, and patient stratification.
Multi‐Parameter Evaluation	Integration of clinical, genetic, and imaging data for holistic decision‐making.
AI Model Performance	Accuracy, reliability, and comparison with traditional decision‐making.
Regulatory & Ethical Issues	Compliance with medical guidelines, transparency, and interpretability challenges.

## 5 CONCLUSION

Utilizing AI in colorectal multimodality practice can enhance patient‐centered care and improve surgical outcomes, thus reducing the incidence and mortality rates. AI can support decision‐making through MDT collaboration and aid in pathology recognition, ultimately reducing workload and minimizing the likelihood of misdiagnosis and missed diagnoses. Embracing AI to optimize decision‐making within MDT reflects a commitment to adopting innovation and application. This approach can objectively provide reliable and comprehensive reference opinions, facilitating more accurate clinical decisions.

## AUTHOR CONTRIBUTIONS

Authors AM and MB were involved in the conceptualization of the manuscript. AM, FK, MJ, SG, and SR were involved in the writing and preparation of the manuscript. Authors F.A.M, IW, MB, and D.M.B were involved in the review and editing of the final draft. All authors read and approved the final submitted version of this manuscript.

## FUNDING INFORMATION

Author D.M.B. is supported by the Royal Society Wolfson Research Fellowship (#WM170007).

## CONFLICT OF INTEREST STATEMENT

D.M.B. is Editor‐in‐Chief of Experimental Physiology, Chair of the Life Sciences Working Group, member of the Human Spaceflight and Exploration Science Advisory Committee to the European Space Agency and member of the Space Exploration Advisory Committee to the UK and Swedish National Space Agencies. D.M.B. is also affiliated to Bexorg, Inc. (USA) focused on the technological development of novel biomarkers of cerebral bioenergetic function and structural damage in humans.

## ETHICS STATEMENT

Ethical approval was not required for this manuscript.

## Data Availability

The evidence used to support this article is publicly available in electronic databases.
